# VvBBX44 and VvMYBA1 form a regulatory feedback loop to balance anthocyanin biosynthesis in grape

**DOI:** 10.1093/hr/uhad176

**Published:** 2023-09-01

**Authors:** Wenwen Liu, Huayuan Mu, Ling Yuan, Yang Li, Yuting Li, Shenchang Li, Chong Ren, Wei Duan, Peige Fan, Zhanwu Dai, Yongfeng Zhou, Zhenchang Liang, Shaohua Li, Lijun Wang

**Affiliations:** Beijing Key Laboratory of Grape Science and Enology and State Key Laboratory of Plant Diversity and Specialty Crops, Institute of Botany, Chinese Academy of Science, Beijing 100093, China; China National Botanical Garden, Beijing 100093, China; University of Chinese Academy of Sciences, Beijing 10049, China; National Key Laboratory of Tropical Crop Breeding, Shenzhen Branch, Guangdong Laboratory of Lingnan Modern Agriculture, Key Laboratory of Synthetic Biology, Ministry of Agriculture and Rural Affairs, Agricultural Genomics Institute at Shenzhen, Chinese Academy of Agricultural Sciences, Shenzhen 518000, China; Beijing Key Laboratory of Grape Science and Enology and State Key Laboratory of Plant Diversity and Specialty Crops, Institute of Botany, Chinese Academy of Science, Beijing 100093, China; China National Botanical Garden, Beijing 100093, China; University of Chinese Academy of Sciences, Beijing 10049, China; Department of Plant and Soil Sciences, Kentucky Tobacco Research and Development Center, University of Kentucky, Lexington, Kentucky 40546, USA; Beijing Key Laboratory of Grape Science and Enology and State Key Laboratory of Plant Diversity and Specialty Crops, Institute of Botany, Chinese Academy of Science, Beijing 100093, China; China National Botanical Garden, Beijing 100093, China; Beijing Key Laboratory of Grape Science and Enology and State Key Laboratory of Plant Diversity and Specialty Crops, Institute of Botany, Chinese Academy of Science, Beijing 100093, China; China National Botanical Garden, Beijing 100093, China; University of Chinese Academy of Sciences, Beijing 10049, China; Beijing Key Laboratory of Grape Science and Enology and State Key Laboratory of Plant Diversity and Specialty Crops, Institute of Botany, Chinese Academy of Science, Beijing 100093, China; China National Botanical Garden, Beijing 100093, China; University of Chinese Academy of Sciences, Beijing 10049, China; Beijing Key Laboratory of Grape Science and Enology and State Key Laboratory of Plant Diversity and Specialty Crops, Institute of Botany, Chinese Academy of Science, Beijing 100093, China; China National Botanical Garden, Beijing 100093, China; Beijing Key Laboratory of Grape Science and Enology and State Key Laboratory of Plant Diversity and Specialty Crops, Institute of Botany, Chinese Academy of Science, Beijing 100093, China; China National Botanical Garden, Beijing 100093, China; Beijing Key Laboratory of Grape Science and Enology and State Key Laboratory of Plant Diversity and Specialty Crops, Institute of Botany, Chinese Academy of Science, Beijing 100093, China; China National Botanical Garden, Beijing 100093, China; Beijing Key Laboratory of Grape Science and Enology and State Key Laboratory of Plant Diversity and Specialty Crops, Institute of Botany, Chinese Academy of Science, Beijing 100093, China; China National Botanical Garden, Beijing 100093, China; National Key Laboratory of Tropical Crop Breeding, Shenzhen Branch, Guangdong Laboratory of Lingnan Modern Agriculture, Key Laboratory of Synthetic Biology, Ministry of Agriculture and Rural Affairs, Agricultural Genomics Institute at Shenzhen, Chinese Academy of Agricultural Sciences, Shenzhen 518000, China; Beijing Key Laboratory of Grape Science and Enology and State Key Laboratory of Plant Diversity and Specialty Crops, Institute of Botany, Chinese Academy of Science, Beijing 100093, China; China National Botanical Garden, Beijing 100093, China; Beijing Key Laboratory of Grape Science and Enology and State Key Laboratory of Plant Diversity and Specialty Crops, Institute of Botany, Chinese Academy of Science, Beijing 100093, China; China National Botanical Garden, Beijing 100093, China; Beijing Key Laboratory of Grape Science and Enology and State Key Laboratory of Plant Diversity and Specialty Crops, Institute of Botany, Chinese Academy of Science, Beijing 100093, China; China National Botanical Garden, Beijing 100093, China

## Abstract

Anthocyanins are essential for the quality of perennial horticultural crops, such as grapes. In grapes, ELONGATED HYPOCOTYL 5 (HY5) and MYBA1 are two critical transcription factors that regulate anthocyanin biosynthesis. Our previous work has shown that *Vitis vinifera* B-box protein 44 (VvBBX44) inhibits anthocyanin synthesis and represses *VvHY5* expression in grape calli. However, the regulatory mechanism underlying this regulation was unclear. In this study, we found that loss of *VvBBX44* function resulted in increased anthocyanin accumulation in grapevine callus. VvBBX44 directly represses *VvMYBA1*, which activates *VvBBX44*. VvMYBA1, but not VvBBX44, directly modulates the expression of grape UDP flavonoid 3-*O*-glucosyltransferase (*VvUFGT*). We demonstrated that VvBBX44 represses the transcriptional activation of *VvUFGT* and *VvBBX44* induced by VvMYBA1. However, VvBBX44 and VvMYBA1 did not physically interact in yeast. The application of exogenous anthocyanin stimulated *VvBBX44* expression in grapevine suspension cells and tobacco leaves. These findings suggest that VvBBX44 and VvMYBA1 form a transcriptional feedback loop to prevent overaccumulation of anthocyanin and reduce metabolic costs. Our work sheds light on the complex regulatory network that controls anthocyanin biosynthesis in grapevine.

## Introduction

Anthocyanins are flavonoid compounds and play important roles in plant function [1–3]. In many perennial fruit crops, the concentration of anthocyanins is highly correlated with the fruit color, which is an important factor in determining their economic value and consumer preference. Anthocyanin biosynthesis is a downstream branch of phenylpropanoid metabolism [4, 5]. FLAVONOID-3-*O*-GLUCOSYLTRANSFERASE (UFGT) catalyzes last step of anthocyanin biosynthesis, transferring glycosyls to flavonoids to generate stable anthocyanins [6, 7]. The MYB-bHLH-WD40 transcription factor (TF) complex (MBW) activates *UFGT* transcription [8, 9]. Many fungible *MYB* genes involved in MBW complexes have been identified in various crops, e.g. *MdMYB1/A/PA1/3/4/6/9/10/11*/*15 L*/*16*/*110a* in apple, *PcMYB10*/*PyMYB10/114*/*PbMYB9/10b* in pear, and *VlMYBA1-1/2/3*, *VlMYBA2*, and *VvMYBA1/2* in grapevine [9–17]. The *ELONGATED HYPOCOTYL 5 (HY5)* gene encodes a basic leucine zipper (bZIP) TF that links light signaling to anthocyanin biosynthesis [18, 19]. Recent research has found that HY5 binds directly to the promoters of *MYBs* in *Arabidopsis*, apple, and pear [10, 20–24]; however, the relationship between HY5 and MYBs still requires further investigation.

B-box (BBX) proteins are a family of TFs that are characterized by the presence of BBX domains. BBX proteins are essential in regulating plant growth and metabolism, particularly in photosignal transduction pathways affecting anthocyanin biosynthesis [25, 26]. In *Arabidopsis*, apple, and pear, a group of BBXs, along with HY5, precisely regulate anthocyanin accumulation through a complex transcriptional regulatory network [23, 27–31]. For instance, in pear, PpBBX16 and PpBBX18 interact with PpHY5 in the PpBBX–PpHY5 complex, which activates the expression of *PpMYB10* and positively regulates anthocyanin biosynthesis [23, 32]. *PpBBX24* mutation reduced *PpMYB10 *and* PpHY5* expression and induced green-skin phenotype [33]. In apple, MdBBX25 functions as a negative regulator of anthocyanin biosynthesis, and its expression is downregulated under UV-B. MdBBX25 attenuates the activation of *MdMYB1* by interacting with MdHY5, and suppresses the expression of *MdANS* and *MdUFGT* by directly binding to their promoters [34]. In contrast, MdHY5 promotes the expression of *MdBBX33* and *MdCOL11*, which in turn modulates the expression of *MdMYBA* to increase anthocyanin accumulation [35]. Several other BBX TFs, MdBBX1/15/17/35/51/54, directly bind to the promoter region of *MdMYB10* to activate its expression, thereby promoting light-induced anthocyanin biosynthesis [37]. Collectively, these observations suggest that BBXs may interact with MYB transcription factors, in addition to HY5, to form a complex transcriptional network that regulates anthocyanin biosynthesis.

Negative feedback loops are common regulatory mechanisms in which the product of a biological system dampens the response to maintain its homeostasis and stability. For example, a plant circadian clock feedback loop incorporating cyclic adenosine diphosphate ribose (cADPR) reduces the impact of environmental changes on circadian rhythms [38, 39]. An miR397b-CASEIN KINASE II SUBUNIT BETA3 (CKB3)-CIRCADIAN CLOCK ASSOCIATED1 (CCA1) feedback circuit regulates flowering time [40]. A characteristic of negative feedback regulation is that the activators and suppressors often have similar expression patterns in anthocyanin biosynthesis, such as AtTT8 (activator) and AtMYBL2 (suppressor) in *Arabidopsis*, PpMYB10.1 (activator) and PpMYB18 (suppressor) in peach, and SlJAF13 (activator) and SlJAZ2 (suppressor) in tomato [41–44].

Grapes (*Vitis vinifera* L. and other *Vitis* species), a widely cultivated and economically important fruit crop, rely on anthocyanins to ensure high-quality wine production and desirable organoleptic properties [16, 45–47]. Grapevine VvBBX44 (VIT_200s0347g00030) contains a BBX domain within its amino-terminal region and shares high similarity to *Arabidopsis* AtBBX28 and AtBBX29, which physically interact with HY5 to inhibit its binding to target gene promoters [19, 30]. However, *VvBBX44* functions differently by directly repressing*VvHY5* transcription in grapevine [48]. In addition, VvMYBA1 directly activates *VvUFGT* expression [6, 49]. The potential relationship between VvBBX44, VvMYBA1, and VvUFGT in anthocyanin biosynthesis is an important question. In this study we aimed to reveal the regulatory mechanism of VvBBX44 in inhibiting anthocyanin biosynthesis. We found that VvBBX44 is an indirect repressor of *VvUFGT* but rather a direct repressor of *VvMYBA1*. VvMYBA1 directly activates *VvUFGT* and *VvBBX44* expression. Although VvBBX44 does not physically interact with VvMYBA1, these two proteins form a feedback regulatory loop to balance anthocyanin biosynthesis in grapevine. Furthermore, the expression of *VvMYBA1* increased earlier than that of *VvBBX44* during berry skin development, and exogenous anthocyanin treatment increased *VvBBX44* expression. Our study highlights the regulatory roles of VvBBX44 and VvMYBA1 in anthocyanin biosynthesis and sheds light on a feedback regulatory loop that balances this process in grapes.

## Results

### VvBBX44 is a nuclear- and cytoplasmic-localized protein likely lacking transcriptional activity

**Figure 1:**
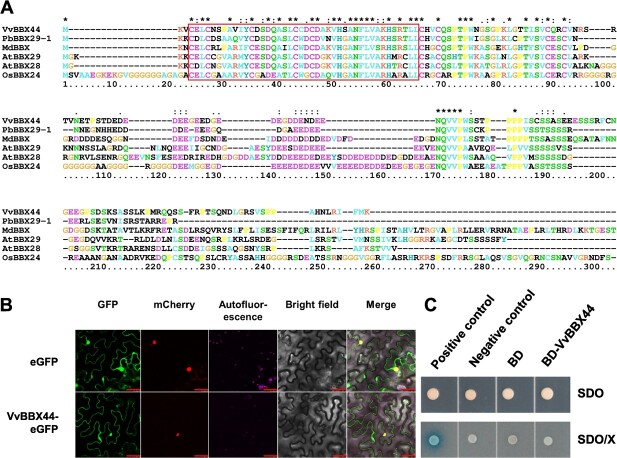
Sequence analysis, subcellular localization, and transcriptional activity of VvBBX44. (A) Deduced amino acid sequence of VvBBX44 is shown aligned with its known homologs: *Vitis amurensis* VaBBX44 (VAG0129707.1), *Pyrus bretschneideri* PbBBX29–1 (LOC103961570), *Malus domestica* MdBBX (MD09G1099500), *Arabidopsis thaliana* AtBBX28 (At4g27310.1) and AtBBX29 (At5g54470.1), and *Oryza sativa* OsBBX24 (Os08t0178800). The B-box (Bbox1_BBX-like, cd19821: 3–45) domain is shown in a box. (B) Subcellular localization of VvBBX44 in tobacco leaves. VvBBX44 fused with enhanced green fluorescent protein (eGFP) and the nuclear localization signal mCherry were transfected into tobacco leaf. GFP fluorescence (green) and RFP fluorescence (red) observed using a confocal microscope are shown. Scale bar is 50 μm. (C) Analysis of VvBBX44 transcriptional activity in yeast. Yeast cells expressing VvBBX44 fused with the Gal4 binding domain (BD) and BD alone were spotted on SD/−Trp (SDO) and SD/−Trp/X-α-Gal (SDO/X) selection media. Transactivation activity was revealed through the expression of the lacZ reporter gene (β-galactosidase activity). BD-Lam and combinations of AD-T with BD-p53 were used as negative and positive controls, respectively.

The 558-bp *VvBBX44* coding sequence (CDS) was amplified from ‘Jingxiu’ (*V. vinifera*) leaf cDNA and sequenced. A single conserved BBX domain is present at VvBBX44 (Fig. 1A). Based on previous classification of grapevine BBX proteins [48], VvBBX44 belongs to Group V of the VvBBX family. A comparison of the amino acid sequence of VvBBX44 with the most closely related proteins from *V. amurensis*, *Pyrus bretschneideri*, *Malus domestica*, *Arabidopsis thaliana*, and *Oryza sativa* revealed strong conservation, particularly within the BBX domain (Fig. 1A). VvBBX44 belongs to the repressor cluster according to the known activators and suppressors in *A. thaliana* and *M. domestica* (Supplementary Data Fig. S1A) [26]. To determine the subcellular localization of VvBBX44, a vector expressing the VvBBX-eGFP fusion was transformed into tobacco leaves. Analysis of the resulting leaves by fluorescence microscopy revealed that VvBBX44 localizes to both nucleus and cytoplasm (Fig. 1B). To investigate the transcriptional activity of VvBBX44, the CDS was fused in-frame with the Gal4 DNA-binding domain, cloned into the pGBKT vector (BD-VvBBX44), and transformed into yeast strain Y2HGold. The combination of activation domain (AD)-T with BD-p53 was used as positive control, and AD-T with BD-Lam served as negative control. The positive control was able to grow on SD/−Trp (SDO) and SD/−Trp/X-α-Gal (SDO/X) media and produced α-galactosidase activity (Fig. 1C). In contrast, yeast cells harboring BD-*VvBBX44*, BD, or the negative control did not exhibit α-galactosidase activity on SDO/X selection medium, suggesting that VvBBX44 does not possess *trans*-activation activity in the yeast system.

### *VvBBX44* knockout increased anthocyanin accumulation in grapevine

**Figure 2:**
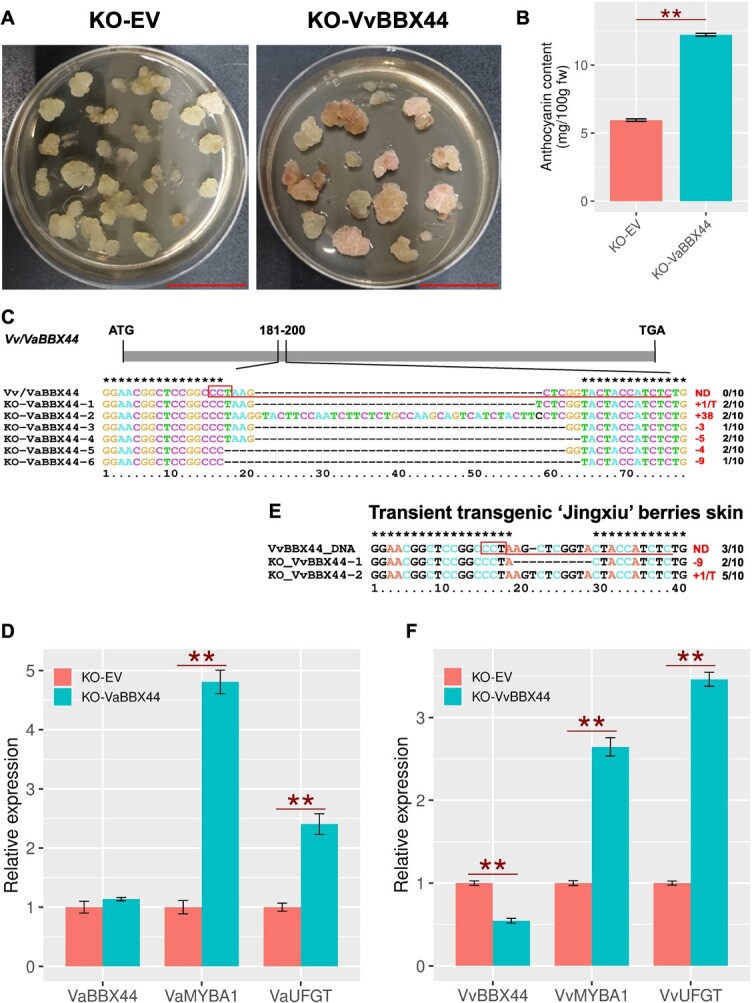
Knockout of *VvBBX44* increased anthocyanin accumulation in grapevine callus and berry skin. The *VvBBX44* sequence was edited by the CRISPR/Cas9 system after transformation into *V. amurensis* petiole and ‘Jingxiu’ berries using the *Agrobacterium*-mediated method. The transgenic petiole was dedifferentiated and regenerated to callus. The transgenic callus was exposed to white light for 7 days before sampling. (A) Phenotypes of KO-EV and KO-*VaBBX44* calli. Scale bar is 3 cm. (B) Anthocyanin concentrations in KO-EV and KO-*VaBBX44* grapevine callus as determined by HPLC analysis. The concentrations were determined according to the characteristic peak area. (C) Identification of *VaBBX44* knockout. The PAM sequence is indicated in a box; target regions are underlined. For each sample, a total of 10 clones were sequenced. The mutation types and numbers of these clones are shown on the right of the diagram, respectively. ND, not detected. Vv/VaBBX44-DNA, *VaBBX44* DNA sequences of control (KO-EV), which the same with *VvBBX44*. KO-VaBBX44, *VaBBX44* knockout sequences. (D) qRT–PCR analysis of *VaBBX44*, *VaMYBA1*, and *VaUFGT* expression in KO-EV and KO-*VaBBX44* callus. (E) Identification of *VvBBX44* knockout mutant. The PAM sequence is indicated by a red box and the target regions are underlined. For each sample, a total of 10 clones were sequenced. The mutation types and numbers of these clones are shown in red and black characters on the right of the diagram, respectively. ND, no mutation detected; *VvBBX44*-DNA, *VvBBX44* DNA sequences of control (KO-EV); KO-*VvBBX44*, *VvBBX44* knockout sequences. (F) qRT–PCR analysis of *VvBBX44*, *VvMYBA1*, and *VvUFGT* expression in *VvBBX44*-knockout and KO-EV berry skin. Gene expression levels are shown relative to EV, which was set to 1. Data are shown as means ± standard errors, which were derived from three replicates. ^**^P < .01; Student’s t-test.

Previously, we demonstrated that overexpression of *VvBBX44* in grapevine callus and leaves downregulates *VvHY5* expression and reduces anthocyanin accumulation [48]. In this study, we used the same experimental samples to investigate the potential effect of *VvBBX44* on *VvMYBA1* expression. The results showed that *VvMYBA1* expression was downregulated in *VvBBX44-*overexpressing cells (Supplementary Data Fig. S1B and C). To evaluate the influence of loss of function of VvBBX44 in anthocyanin biosynthesis, we used *V. amurensis* callus, which can synthesize anthocyanins under light, as the experimental material. It is worth noting that VvBBX44 and VaBBX44 from *V. amurensis* share 98.38% sequence similarity with the same BBX domain (Fig. 1A). We disrupted the *VaBBX44* gene in the same target sequence as *VvBBX44* using the CRISPR-Cas9 system, and produced six distinct mutations around the same PAM site, including both deletion and insertion, which would result in frameshift and premature translation termination (Fig. 2C and E). After 7 days of white light treatment, several of the *VaBBX44*-knockout calli (KO*-VaBBX44*) had developed a light pink color (Fig. 2A). HPLC analysis revealed a higher anthocyanin content in the KO*-VaBBX44* callus compared with control callus transformed with the empty vector (KO-EV) (Fig. 2B). In the absence of light, no obvious color development was observed in the KO-*VaBBX44* callus (Supplementary Data Fig. S2B). Moreover, *VaBBX44* knockout significantly (*P* < .01) increased the expression of *VaMYBA1* and *VaUFGT* (Fig. 2D). We also knocked out *VvBBX44* in berry skin tissue of the *V. vinifera* cultivar ‘Jingxiu’ using the CRISPR-Cas9 system*.* After 5 days of white light treatment, the expression levels of *VvMYBA1* and *VvUFGT* were higher in the KO-*VvBBX44* berry skin than in the skin of KO-EV berry (Fig. 2E and F). These results further suggest that VvBBX44 represses expression of *VvMYBA1* and *VvUFGT*.

### VvBBX44 directly suppresses *VvMYBA1* expression

**Figure 3:**
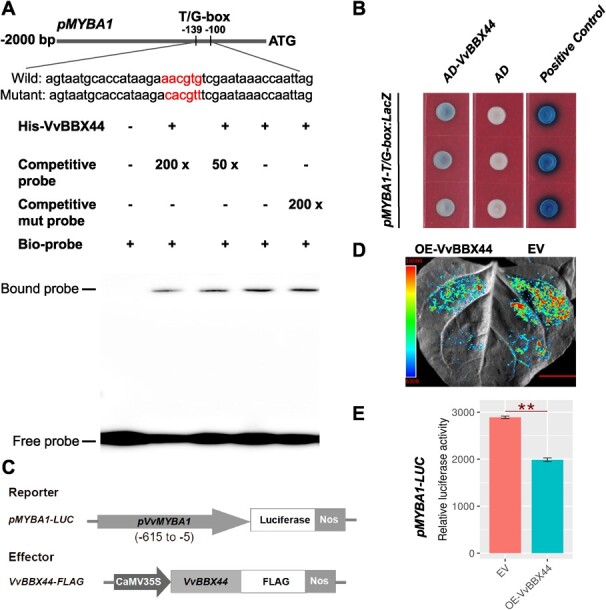
VvBBX44 directly suppresses *VvMYBA1* expression. (A) EMSA showing the binding of VvBBX44 to the *VvMYBA1* promoter containing the T/G-box. Non-labeled wild-type or mutant probes were added as competitors. (B) Y1H assays of VvBBX44 binding to the *VvMYBA1* promoter containing the T/G-box. The full-length *VvBBX44* CDS was fused to the pGAD424 activation domain (AD), and the resulting plasmid (AD-*VvBBX44*) was co-transformed with the pMYBA1-T/G-box:LacZ reporter into yeast cells. The transformants were further grown on SD/−Trp/−Leu/−Ura selection medium supplied with 80 mg L^−1^ X-Gal for color development. AD-VvMYB14 + pVvSTS15/21:LacZ was the positive control. (C, D, E) Luciferase activity analysis. (C) Schematic diagram of reporter and effector constructs. The full-length *VvBBX44* CDS was inserted into the pSAK277 vector to generate the effector construction. The truncations of the *VvMYBA1* promoter were cloned into the vector pCAMBIA1302-LUC to generate reporter constructs (pMYBA1-LUC). (D) pMYBA1-LUC in the presence or absence of *VvBBX44* effector were transfected into tobacco leaves. The empty vector (EV) combined with pMYBA1-LUC was used as the control. In (D) and (E) luciferase activity was measured and data are shown as means ± standard errors, which were derived from three replicates. ^**^P < .01; Student’s *t*-test. Scale bar is 1 cm. The experiments were performed three times with similar results, and a representative image is shown.

To investigate whether VvBBX44 directly regulates *VvMYBA1* expression, we examined the binding of VvBBX44 to the *VvMYBA1* promoter. Previous studies in apple have shown that BBXs bind to the T/G-box (CACGTT) element of in the *MdMYB1* promoter [34, 50]. In this work, we cloned the *VvMYBA1* promoter (*pVvMYBA1*) and identified a single T/G-box element ~120 bp upstream of the start codon (Fig. 3A and Supplementary Data 1). An electrophoretic mobility shift assay (EMSA) was performed and revealed that VvBBX44 bound to the probe containing the T/G-box sequence (Fig. 3A). We also conducted yeast one-hybrid (Y1H) assays using the same short oligonucleotide containing this T/G-box sequence as that used in EMSA (Fig. 3A), and revealed that VvBBX44 binds to the *VvMYBA1* promoter containing the T/G-box. The luciferase reporter assay showed that VvBBX44 suppressed the activity of *proVvMYBA1* (*pMYBA1*) (Fig. 3C–E). These findings suggest that VvBBX44 negatively regulates *VvMYBA1* expression by directly binding to its promoter.

### Expression of *VvBBX44* and *VvMYBA1* during berry development and coloring

**Figure 4:**
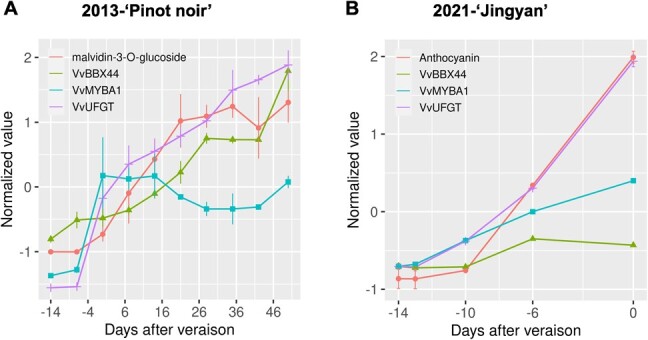
Expression of *VvBBX44*, *VvMYBA1*, and *VvUFGT* and malvidin-3-*O*-glucoside content during berry coloring. (A) ‘Pinot Noir’ berry in 2013. Expression data are from a published RNA-seq analysis (accession number GSE98923). (B) ‘Jingyan’ berry in 2021. 0 represents veraison.

The results of this study, along with previous literature [6, 48, 49], suggest that VvBBX44 suppresses while VvMYBA1 promotes anthocyanin biosynthesis. To understand how the expression levels of *VvBBX44* and *VvMYBA1* change during grape berry development and coloring, we examined publicly available RNA-seq data from developing berry of *V. vinifera* ‘Pinot noir’ (accession GSE98923) (Supplementary Data Fig. S3) [51]. As shown in Fig. 4A, the expression levels of both *VvBBX44* and *VvMYBA1* increased before veraison, with *VvMYBA1* expression increasing earlier and more rapidly than *VvBBX44* expression. After veraison, *VvMYBA1* expression was maintained at a relatively high level before gradually decreasing, while expression of *VvBBX44* continued to increase. To verify this finding, we analyzed the expression patterns of *VvBBX44* and *VvMYBA1* in berries of *V. vinifera* ‘Jingyan’ before veraison and observed a similar trend (Fig. 4B). These results suggest that VvMYBA1 regulates the expression of *VvBBX44*.

### VvMYBA1 directly activates *VvBBX44* expression

**Figure 5:**
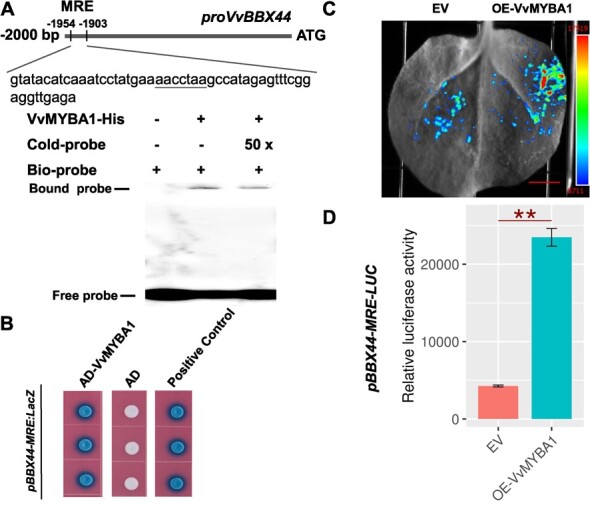
VvMYBA1 directly activates *VvBBX44* expression. (A) EMSA analysis showing binding of VvMYBA1 to the *VvBBX44* promoter containing the MRE motif. The location of the MRE motif is underlined. A 50-fold excess of unlabeled probe was added as the competitor. The experiments were performed three times with similar results, and a representative image is shown. (B) Y1H assays of VvMYBA1 binding to the *VvBBX44* promoter. The full-length *VvMYBA1* CDS was fused to the pGAD424 activation domain (AD), and the resulting plasmid (AD-VvMYBA1) was co-transformed with the pBBX44-MRE:LacZ reporter into yeast cells. The transformants were further grown on SD/−Trp/−Leu/−Ura selection medium supplied with 80 mg L^−1^ X-Gal for color development. AD-VvMYB14 + pVvSTS15/21:LacZ was the positive control. (C and D) Luciferase activity analysis. The full-length *VvMYBA1* CDS was fused to the pSAK277 vector to generate the effector construction. The fragment of the *VvBBX44* promoter harboring the MRE element was cloned into the vector pCAMBIA1302-LUC to generate the reporter construct pBBX44-MRE-LUC. pBBX44-MRE-LUC in the presence or absence of VvMYBA1 effector was infiltrated into tobacco leaves. The empty vector (EV) + pBBX44-MRE-LUC was used as the control. Data are shown as means ± standard errors, which were derived from three replicates. ^**^P < .01; Student’s *t*-test. In (C) the scale bar represents 1 cm.

We next analyzed potential *cis*-acting elements in the *VvBBX44* promoter and found one MYB-recognition element (MRE), which is known to be involved in light response, located 1926 bp upstream of the ATG (Fig. 5A)*.* Results of a Y1H assay indicated that VvMYBA1 can bind to this *pBBX44-MRE* (Fig. 5B). Furthermore, a luciferase reporter assay demonstrated that VvMYBA1 increased the activity of *pBBX44-MRE*-LUC (Fig. 5C and D). Subsequently, we examined whether VvMYBA1 directly binds to the MRE element using EMSA (Fig. 5A). The results revealed that recombinant VvMYBA1 protein physically interacts with the *VvBBX44* promoter containing the MRE.

To further support a role for VvMYBA1 as a positive regulator of *VvBBX44*, we examined the expression of *VvBBX44* in both stable transgenic suspension cells and transiently transformed ‘Jingxiu’ berry skin overexpressing *VvMYBA1* (OE-*VvMYBA1*). The results showed that the expression levels of *VvBBX44* and *VvUFGT* were significantly higher in *VvMYBA1*-overexpressing suspension cells and berry skin under white light condition compared with the EV controls (Supplementary Data Fig. S4C and E). Moreover, we found that *VvMYBA1*-overexpressing grapevine suspension cells turned red (Supplementary Data Fig. S4A and B). The anthocyanin content was significantly (*P* < .01) higher in OE-*VvMYBA1* ‘41B’ grape suspension cells and ‘Jingxiu’ berry skin than in the corresponding EV controls (Supplementary Data Fig. S4D and F).

### VvBBX44 represses the transcriptional activity of VvMYBA1 on *VvUFGT* and *VvBBX44*

We demonstrated that VvBBX44 acts as a negative regulator for anthocyanin biosynthesis, potentially by inhibiting the transcription of VvMYBA1. However, it is not clear if VvBBX44 directly interacts with VvMYBA1 to inhibit its activity. In the STRING analysis results (https://string-db.org/), there was no evidence of a direct interaction between VvBBX44 and VvMYBA1 (Supplementary Data Fig. S5A). To investigate the potential interaction between VvMYBA1 and VvBBX44, we used the yeast two-hybrid (Y2H) assay. The full-length CDSs of *VvMYBA1* and *VvBBX44* were subcloned into the pGADT7 vector and pGBKT7 vector, respectively. Yeast transformants harboring AD-VvMYBA1/BD-VvBBX44 were able to grow on SD/−Leu/−Trp selection medium, but not on SD/−Leu/−Trp/−His/−Ade selection medium supplied with 40 mg L^−1^ X-α-Gal and 200 ng mL^−1^ AbA (Supplementary Data Fig. S5B), indicating that VvMYBA1 does not physically interact with VvBBX44 in yeast.

**Figure 6:**
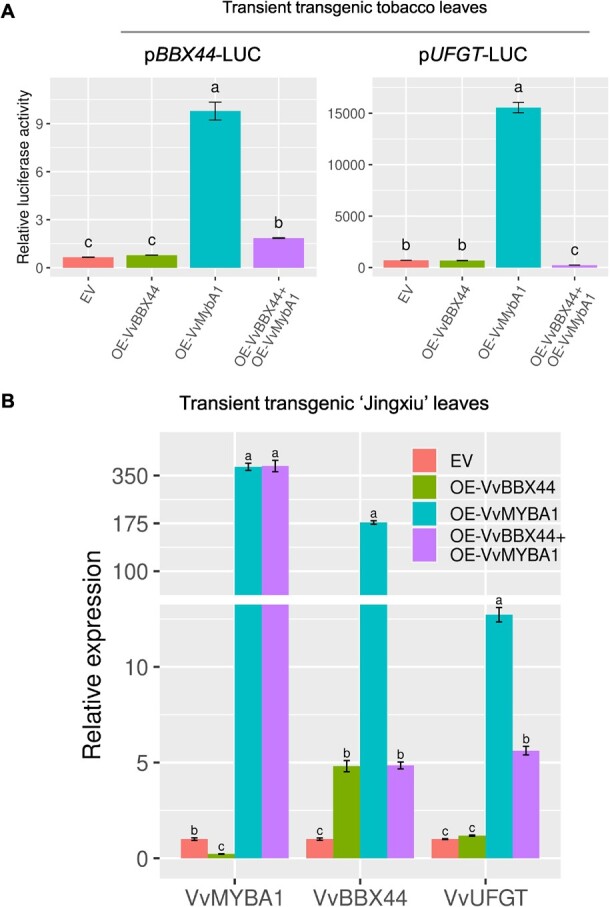
VvBBX44 inhibits transcriptional activation of *VvBBX44* and *VvUFGT* by VvMYBA1. (A) VvBBX44 effects on the transcriptional activation of pBBX44-LUC and pUFGT-LUC reporter by VvMYBA1 in tobacco leaves. The pBBX44-LUC reporter, VvMYBA1 effector, and/or VvBBX44 effector were co-transformed into tobacco leaves. The pUFGT-LUC reporter, VvMYBA1 effector, and/or VvBBX44 effector were co-transformed into tobacco leaves. EV represents the controls used for the pBBX44-LUC or pUFGT-LUC reporter with the EV effector. Different letters above the bars indicate significant differences according to Duncan’s test (*P* < .01). (B) Expression of *VvMYBA1*, *VvBBX44*, and *VvUFGT* in ‘Jingxiu’ leaves transiently transformed with constructs overexpressing *VvBBX44* (OE-VvBBX44) alone, *VvMYBA1* (OE-VvMYBA1) alone or co-overexpressing both *VvBBX44* and *VvMYBA1*. Gene expression levels are shown relative to EV, which was set as 1. Data are shown as means ± standard errors, which were derived from three replicates.

To further investigate the effect of VvBBX44 on the transcriptional activation activity of VvMYBA1, we conducted additional experiments. We performed the luciferase reporter assay and found that *VvMYBA1* overexpression alone significantly (*P* < .01) enhanced the transcriptional activity of the *VvBBX44* and *VvUFGT* promoters by VvMYBA1. However, co-overexpression of *VvBBX44* and *VvMYBA1* reduced the activation of *VvBBX44* and *VvUFGT* expression by VvMYBA1 in tobacco leaves (Fig. 6A). These results suggest that VvBBX44 alone has no effect on the activity of its own promoter or the *VvUFGT* promoter. We further examined ‘Jingxiu’ grapevine leaves transiently overexpressing individual *VvMYBA1* (OE*-VvMYBA1*) or *VvBBX44* (OE*-VvBBX44*)*,* or both *VvMYBA1* and *VvBBX44* (OE*-VvMYBA1* + OE*-VvBBX44*) by agroinfiltration. We selected ‘Jingxiu’ leaf samples with similar expression levels of *VvMYBA1* in OE*-VvMYBA1* and OE*-VvMYBA1* + OE*-VvBBX44*, and those with similar expression levels of *VvBBX44* in OE*-VvBBX44* and OE*-VvMYBA1 +* OE*-VvBBX44*. The results showed that the expression levels of *VvBBX44* and *VvUFGT* were significantly (*P* < .01) higher in OE*-VvMYBA1* than in EV, but significantly (*P* < .01) lower in OE*-VvMYBA1* + OE*-VvBBX44* than in OE*-VvMYBA1* (Fig. 6B)*.* Together, these finding suggest that VvBBX44 interferes with the transcriptional activation of VvMYBA1 on *VvBBX44* and *VvUFGT in vivo*.

### Exogenous anthocyanin induces the expression of *VvBBX44*

**Figure 7:**
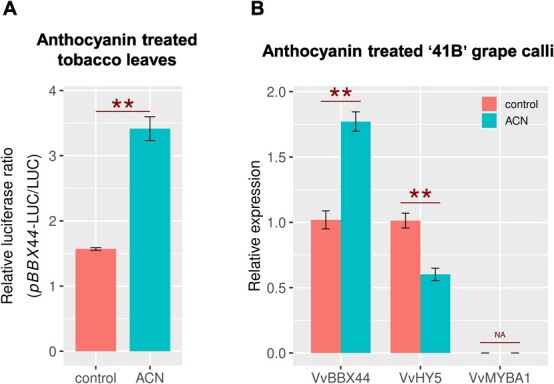
Exogenous anthocyanin (ACN) induces the expression of *VvBBX44*. (A) Effect of exogenous anthocyanin on the activity of pBBX44-LUC in tobacco leaves. *Agrobacterium*-harboring plasmids with the *VvBBX44* promoter region driving expression of the LUC reporter gene were suspended in 1.34 × 10^–6^ mol L^−1^ cyanidin-3-glucoside, and injected into tobacco leaves. (B) Effect of exogenous anthocyanin on the expression of *VvBBX44*, *VvHY5*, and *VvMYBA1* in ‘41B’ suspension cells. Cyanidin-3-glucoside (6.7 × 10^−7^ mol L^−1^) was added to the culture medium and calli were subcultured for 24 h under white light. Expression levels of genes were normalized to *VvACTIN7* and are relative to the control value, which was set to 1. Data are shown as means ± standard errors, which were derived from three replicates. ^**^P < .01; Student’s *t*-test. Since *VvMYBA1* expression was not detected a significance test was not available (NA).

We observed that during grape berry coloring, the accumulation of anthocyanin coincided with an increase in the expression of *VvBBX44* (Fig. 4A and Supplementary Data Fig.S3), thus hypothesizing that anthocyanin may induce the expression of *VvBBX44.* To test this hypothesis, we evaluated the effect of anthocyanin on the activity of the *VvBBX44* promoter. A vector expressing the luciferase (LUC) reporter gene under control of the *VvBBX44* promoter was infiltrated into tobacco leaves with or without anthocyanin cyanidin-3-glucoside. We conducted a preliminary test and determined that a concentration of 1.34 × 10^−6^ mol L^−1^ of cyanidin-3-glucoside only inhibited tobacco leaf growth, while 2.14 × 10^−5^ mol L^−1^ resulted in the death of tobacco leaves. The anthocyanin treatment at 1.34 × 10^−6^ mol L^−1^ resulted in higher LUC activity compared with the control (water) in tobacco leaves (Fig. 7A), suggesting that the *VvBBX44* promoter activity was enhanced in the presence of exogenous anthocyanin. To further investigate the relationships between anthocyanin and *VvBBX44* expression, we evaluated the effects of exogenous cyanidin-3-glucoside on *VvMYBA1* and *VvBBX44* expression in ‘41B’ suspension cells. Compared with the control, anthocyanin treatment significantly increased expression of *VvBBX44*. However, we were unable to detect the expression of *VvMYBA1* in ‘41B’ suspension cells, with or without exogenous anthocyanin. Nonetheless, we observed that the expression of *VvHY5* was significantly reduced with anthocyanin treatment (Fig. 7B).

## Discussion

### VvBBX44 is a repressor of anthocyanin biosynthesis in grape

BBX proteins play important roles in regulating light-induced anthocyanin biosynthesis in *Arabidopsis*, apple, and pear [30]. However, little is known about the regulation of anthocyanin biosynthesis by BBXs in grapevine. Recently, we reported that VvBBX44 directly suppresses *VvHY5* expression and anthocyanin biosynthesis in grapevine [48]. Although *Arabidopsis* AtBBX28 and AtBBX29 are most closely related to VvBBX44 (Fig. 1A), they do not directly suppress *HY5* expression; instead, they interfere with the binding of HY5 to the promoters of HY5-targeted genes through physical interaction, thus repressing HY5 transcriptional activity [19, 30]. Under light conditions, the anthocyanin content in *VvBBX44*-overexpressing callus is lower than that of EV control callus [48]. In this study, we found that *VvBBX44*-knockout grape callus had higher anthocyanin content than EV callus under light conditions (Fig. 2A–C). We also discovered that VvMYBA1 activates the *VvUFGT* promoter*,* while this activation was strongly suppressed when *VvBBX44* was co-expressed with *VvMYBA1* (Fig. 6). These results collectively indicate that VvBBX44 inhibits anthocyanin biosynthesis under light.

### VvBBX44 controls anthocyanin biosynthesis through feedback regulation

Negative feedback loops for anthocyanin biosynthesis pathways have been identified in various crop plants. The negative feedback loops differ between plants. For example, in apple, mdm-miR828 is positively correlated with anthocyanin concentration and can be induced by MdMYB1, which inhibits anthocyanin biosynthesis by increasing degradation of MdMYB1 [52]. In pear, *PpMYB18* expression is triggered by anthocyanin and proanthocyanin during ripening and juvenile stages, competing with MYB activators for interacting with a basic helix–loop–helix (bHLH) TF to reduce anthocyanin biosynthesis [43]. *Brassica napus* BnCPC is an R3-MYB TF, and *BnCPC* expression is activated by the MBW complex. BnCPC in turn competes with BnPAP1 of the MBW complex and inhibits anthocyanin biosynthesis [53]. In these feedback loops, the repressors are induced by either anthocyanin or their transcriptional activators. The repressors then inhibit the expression of anthocyanin biosynthetic regulators or interrupt MBW complex formation to repress anthocyanin accumulation. Anthocyanin pathway repressors have been identified in grape. VvMYBC2L2 and VvMYB86 repress grape anthocyanin biosynthesis. VvMYBC2L2 inhibits anthocyanin accumulation in grapevine tissues [54], while VvMYB86 inhibits the conversion of proanthocyanin to anthocyanins [55]. However, VvMYBC2L2 and VvMYB86 are negatively correlated with anthocyanin and do not form feedback loops for anthocyanin biosynthesis in grape. In this study, we demonstrated that VvBBX44 is an anthocyanin biosynthesis repressor. Its expression levels were positively correlated with anthocyanin concentrations in grape berry skin (Fig. 4), and it is induced by anthocyanin while repressing *VvMYBA1* expression. These findings suggest that VvBBX44 acts as a negative feedback regulator for grape anthocyanin biosynthesis.

### VvBBX44 represses *VvMYBA1*

This study revealed that the expression of both *VvBBX44* and *VvMYBA1* increased during veraison (Fig. 4A), consistent with the finding that the expression of VvMYBA1 positively regulates *VvUFGT* expression [6, 49]. Previous studies have also reported that activators (CsRuby1 and PpMYB10.1) and repressors (CsMYB3 and PpMYB18) co-express in anthocyanin-accumulating tissues in citrus (*Citrus* spp.) and peach (*Prunus persica*) [41, 43, 56]. Mu *et al*. showed that the repressors VvWRKY8 and VvMYB30, as well as the activator VvMYB14, co-express in grape berry skin, where resveratrol is predominantly accumulated [57]. Therefore, concurrent expression of both activators and repressors is a hallmark of specialized metabolic pathways. The relationship between the repressor VvBBX44 and the activator VvMYBA1, as well as HY5, warrants further investigation.

The question we posed is: how does VvBBX44 act as a repressor of anthocyanin biosynthesis? Regulatory relationships between BBXs and MYBs have been reported previously. For example, in apple, MdBBX25 does not bind to the promoter of *MdMYB1* but reduces the positive effect of MdHY5 on *MdMYB1* by interacting with MdHY5 [34]. In pear, PpBBX16 and PpBBX18 do not directly bind to the promoter of *PpMYB10*, but can enhance the promoter activity of *PpMYB10* [23, 32]. MdBBX20 can directly bind the *MdMYB1* promoter region and activates *MdMYB1* expression [36]. In this study, we demonstrated that VvBBX44 directly binds to the *VvMYBA1* promoter and inhibits its expression (Fig. 3). VvBBX44 has been shown to repress *VvHY5* expression [48]. In addition, in pear, PpHY5 and PyHY5 bind to the promoters of *PpMYB10* and *PyMYB10* (homolog of VvMYBA1), respectively, and activate their expression [23, 24]. Taken together, these results suggest that VvBBX44 represses *VvMYBA1* either directly or indirectly via VvHY5. However, the expression levels of both *VvMYBA1* and *VvBBX44* increased prior to veraison, with *VvMYBA1* expression increasing relatively early (Fig. 4). This finding suggests that VvMYBA1 may induce the expression of *VvBBX44*. We analyzed the *VvBBX44* promoter sequence for potential *cis*-acting sequences and identified an MYB-binding motif involved in light response (Fig. 5A). Our experiments confirmed that VvMYBA1 binds to the MYB-binding site in the *VvBBX44* promoter and induces its expression (Fig. 5 and Supplementary Data Fig. S4C and E). Collectively, these results suggest that VvBBX44 represses *VvMYBA1* expression either directly or indirectly via VvHY5, while VvMYBA1 directly induces *VvBBX44* expression (Figs 3 and5).

*UFGT* is a key gene in anthocyanin biosynthesis. In apple, MdBBX25 directly binds to the promoters of *MdUFGT*s to repress their expression [34]. In this study, we determined that VvBBX44 does not affect the promoter activity of *VvUFGT* but reduces the activation of VvUFGT expression by VvMYBA1 (Fig. 6). This is similar to the effect of MdBBX37 on *MdUFGT* in apple [28]. MdBBX37 does not directly bind to the *MdUFGT* promoter but reduces *MdUFGT* promoter activation by MdMYB1. In grapevine, *VvMYBA1* overexpression activated the expression of *VvUFGT* (Supplementary Data Fig. S4C and E), but co-overexpression of *VvBBX44* and *VvMYBA1* attenuated this activation (Fig. 6). Our results indicated that VvBBX44 indirectly represses *VvUFGT* expression not only by repressing *VvMYBA1* expression but also by interfering with the transcriptional activity of VvMYBA1. However, we did not detect physical interaction of VvBBX44 with VvMYBA1 in yeast (Supplementary Data Fig. S5). We inferred that VvBBX44 inhibits the effect of VvMYBA1 by forming protein complex(es) with additional TF(s). The inter-regulatory and similar expression patterns between *VvBBX44* and *VvMYBA1* suggest that they provide feedback regulation upon anthocyanin biosynthesis.

### VvBBX44 and VvMYBA1 form a feedback regulatory loop to balance anthocyanin biosynthesis in grape

Multiple lines of evidence support the important roles of VvBBX44 and VvMYBA1 in anthocyanin synthesis: (i) the expression of *VvBBX44* is induced by VvMYBA1 and anthocyanin (Figs 5, 6B and 7; Supplementary Data Fig. S4); (ii) VvBBX44 binds to the promoters of *VvMYBA1* and *VvHY5* to represses their expression, while VvHY5 induces *VvMYBA1* expression (Supplementary Data Figs S1B and C andS6; Figs 2D and F and3) [48]; (iii) VvBBX44 interferes with the activation of *VvUFGT* and *VvBBX44* by VvMYBA1 (Fig. 6). However, VvBBX44 has no direct effect on *VvUFGT* expression.

**Figure 8:**
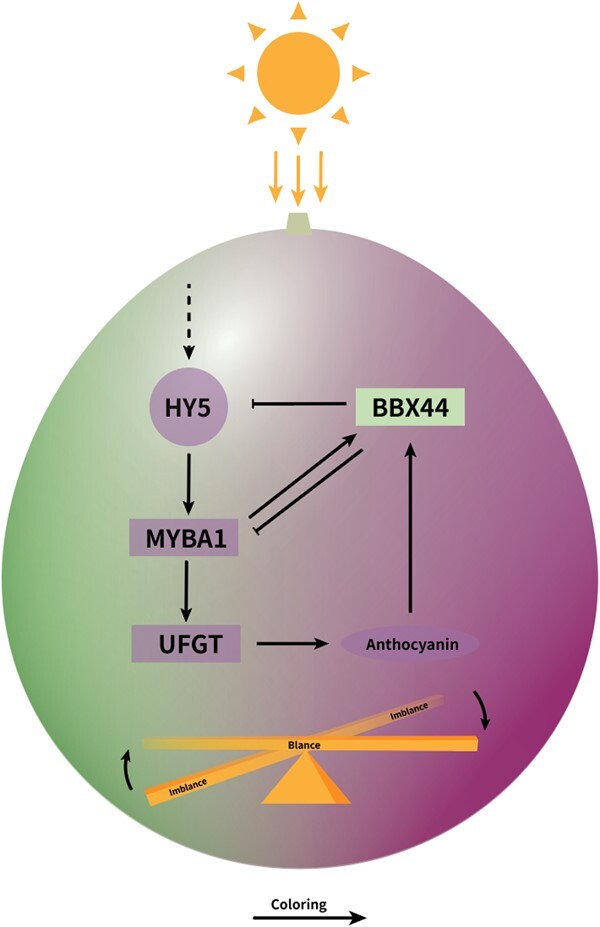
A model for the BBX44-MYBA1 regulatory loop controlling anthocyanin biosynthesis in grapevine berry. When berries are exposed to light, *VvMYBA1* is activated by VvHY5, promoting the transcription of *VvUFGT*, and anthocyanin biosynthesis is stimulated. At the same time, VvMYBA1 activates the transcription of *VvBBX44*. When the anthocyanin concentration reaches a threshold level, it also induces the transcription of VvBBX44. In turn, VvBBX44 directly represses the transcription of *VvMYBA1* and *VvHY5* and *VvUFGT* expression is decreased, thus resulting in a balance of anthocyanin concentration.

Based on our evidence, we propose a model for the feedback regulation of grape anthocyanin biosynthesis centered on VvBBX44 and VvMYBA1 (Fig. 8). When berries are exposed to light, VvHY5 activates *VvMYBA1*, which induces the expression of *VvUFGT*, leading to increased anthocyanin biosynthesis. Simultaneously, VvMYBA1 induces the expression of *VvBBX44*. When the anthocyanin concentration reaches a certain level, it activates *VvBBX44* expression. VvBBX44 then directly represses the expression of *VvMYBA1* and *VvHY5*, resulting in decreased *VvUFGT* expression and a dynamic balance of anthocyanin concentration. Therefore, the VvBBX44–VvMYBA1 regulatory loop serves as an important mechanism for the fine-tuning of anthocyanin biosynthesis in grapevine. This mechanism would prevent overaccumulation of anthocyanin to save metabolic costs while providing appropriate photoprotection. Our work expanded our knowledge on the regulatory network for anthocyanin biosynthesis in grapevine.

## Materials and methods

### Plant materials, growth conditions, and treatments

*Vitis vinifera* ‘Jingxiu’ and ‘Jingyan’ were obtained from the Germplasm Repository for Grapevines at the Institute of Botany, Chinese Academy of Sciences, Beijing, China (39°54′N, 116°23′E). Healthy leaves ~20 days after emergence and berries at 14 days before veraison of ‘Jingxiu’ were used for transformation. ‘Jingyan’ was used to analyze *VvBBX44* and *VvMYBA1* expression during berry development. Micropropagated *V. amurensis* plant materials were grown on half of the full concentration Murashige and Skoog (MS) medium with a pH value of 5.8 as previously described [58]. Six-week-old plantlets were used for transgenic callus induction.

‘41B’ (*V. vinifera* ‘Chasselas’ × *V. berlandieri*) embryogenic suspension cells were cultured as described previously [59]. Tobacco (*Nicotiana benthamiana*) was grown in a growth chamber with a 16-h light/8-h dark photoperiod, 70–75% relative humidity, and a stable temperature of 23°C. Light treatments were performed in a growth chamber at 25°C with constant illumination of 100 μmol m^−2^ s^−1^ provided by LEDs. For anthocyanin treatment of leaves, *Agrobacterium tumefaciens* harboring a *VvBBX44* promoter:LUC reporter gene were suspended in 6.7 × 10^−7^, 1.34 × 10^−6^, 2.68 × 10^−6^, 5.36 × 10^−6^, 1.07 × 10^−5^, or 2.14 × 10^−5^ mol L^−1^ cyanidin-3-glucoside, and injected into tobacco leaves. For anthocyanin treatment of suspension cells, 6.7 × 10^−7^ mol L^−1^ cyanidin-3-glucoside was added to the culture medium and cells were maintained for 24 h in the light.

### Sequence cloning and analysis

The Super Plant Genomic DNA extraction Kit (Tiangen, China, Cat: DP360) was used to isolate total genomic DNA from samples. The Column Plant RNAout2.0 (Tiangen, China, Cat: DP441) was used to extract total RNA from samples. The extracted total RNA was used for synthesis of the first-strand cDNA using the HiScript II 1st Strand cDNA Synthesis Kit (Vazyme, China, Cat: R212-01). The CDSs of *VvBBX44*, *VvHY5*, and *VvMYBA1* were amplified from ‘Jingxiu’ leaf cDNA, using I5™ High-Fidelity DNA Polymerase (Cat: TP001, Tsingke, China). Promoter segments of *VvBBX44, VvMYBA1*, and *VvUFGT* were amplified from ‘Jingxiu’ leaf DNA. The amplified segments were cloned into *Escherichia coli* TOP10 strain via pLB simple vector (Tiangen, China, Cat: R045A) for sequencing. Sequences were aligned using DNAMAN 7.0.

### Quantitative real-time PCR analysis

cDNA for qRT–PCR was synthesized by using Vazyme HiScript II Q RT SuperMix for qRT–PCR (China, Cat: R223-01). *VvACTIN-7* (VIT_04s0044g00580) was used as reference. There were three biological replicates for each experiment, which comprised three technical replicates, respectively. The relative expression was computed by the Δ–Δ method [60]. All qRT–PCR primers are listed in Supplementary Data Table S1.

### Transformation of ‘41B’ grape suspension cells and *V. amurensis* petiole

The full-length *VvMYBA1* with homologous joints (*VvMYBA1*-eGFP, Supplementary Data Table S1) was ligated into pCambia2300-eGFP. The empty and *VvMYBA1* vectors were transformed into ‘41B’ callus by *A. tumefaciens*-mediated transformation [61] to produce EV and *VvMYBA1*-OE, respectively. Positive transformations were confirmed by GFP signal detection and qRT–PCR. Three independent transgenic lines were selected for further analyses.

To construct the *VvBBX44* knockout vector by the use of CRISPR/Cas9, the genomic DNA sequence of *VvBBX44* was obtained from URGI (Unité de Recherche Génomique Info), and the conserved sequence of *VvBBX44* was used to design a pair of complementary primers (*VvBBX44*_sgRNA_target) with Geneious (Bioinformatics Software for Sequence Data Analysis). The complementary primers were annealed and ligated to the plasmid vector pKSE402 containing eGFP, the Cas9 nuclease and the small guide RNA (sgRNA). The recombinant plasmid containing *VvBBX44* (pKSE402-*VvBBX44*) was confirmed by sequencing and transferred into *A. tumefaciens* (strain EHA105) by the freeze–thaw method. *Agrobacterium tumefaciens* cells harboring pKSE402 and pKSE402-*VvBBX44* were used for the transformation of *V. amurensis* petioles as described by Zhao *et al*. [58], and successful transformation was monitored by GFP fluorescence. To verify the positive gene editing results, the target regions for sgRNA were amplified and sequenced.

### Yeast assays

For VvBBX44 transcriptional activity assays, the *VvBBX44* CDS was fused to the GAL4 BD. The resulting construct (BD-*VvBBX44*) was transferred to yeast strain Y2HGold. Combination of AD-T with BD-p53 was used as positive control, and AD-T with BD-Lam was used as negative control. Transformants were selected on SD/−Trp media, and *trans*-activation activity of VvBBX44 was evaluated by monitoring X-α-Gal (5-bromo-4-chloro-3-indoxyl-α-d-galactopyranoside) on SD/−Trp/X-α-Gal selection media.

The combination activities of VvMYBA1 to MRE motif of *VvBBX44* promoter and VvBBX44 to T/G-box of *VvMYBA1* promoter were examined by a Y1H assay as previously described [62]. Briefly, the *VvBBX44* promoter fragment harboring the MRE and *VvMYBA1* promoter fragment harboring the T/G-box were cloned into the pLacZi vector, forming *pBBX44-MRE:LacZ* and *pMYBA1-T/G-box:LacZ*, respectively. The full-length CDSs of *VvMYBA1* and *VvBBX44* were ligated into the EcoRI-XhoI sites of the pGAD424 vector (Clontech) to generate the prey vector (AD-*VvMYBA1* and AD-*VvBBX44*). Plasmids for AD fusions were each co-transformed with LacZ reporter constructs into the EGY48 yeast strain. Transformed yeast was grown on SD/−Trp−Ura dropout plates containing X-Gal (5-bromo-4-chloro-3-indoxyl-β-d-galactopyranoside) for the blue color reaction.

The *VvMYBA1* promoter fragments (extending from 808 to 5 bp upstream of the ATG start codon, and 321 to 5 bp upstream of the start codon) were ligated into vector pAbAi (Clontech, USA) digested with restriction endonucleases HindIII and XhoI (New England Biolabs, USA), to form *pAbAi-VvMYBA1* and *pAbAi-T/G-box*, respectively. The *VvHY5* full-length cDNA was inserted into vector pGADT7 (Clontech) by homologous recombination. All fragments cloned into vectors were detected by PCR and sequenced to verify. Assays were performed using the Matchmaker One-Hybrid Library Constructions & Screening Kit (Clontech) following the manufacturer’s protocols. The positive control detected interaction between vectors 53-AbAi and pGADT7 (Clontech) and the negative control interaction between vectors pAbAi and pGADT7. SD/−Ura/AbA (Aureobasidin A) medium (materials purchased from Clontech) with an AbA concentration of 250 ng mL^−1^ was used to select for AbA resistance, which is activated by proteins that specifically interact with this DNA sequence.

The system used to perform the Y2H assay was purchased from Clontech. The *VvMYBA1* and *VvBBX44* CDSs were subcloned in-frame into the activation domain (AD) vector pGADT7 and the DNA-binding domain (BD) vector pGBKT7, respectively. Positive colonies were plated onto SD/−Leu/−Trp/−His/−Ade/AbA selection media supplemented with 40 mg L^−1^ X-α-Gal. Combinations of AD-T with BD-p53 and BD-Lam served as positive and negative controls, respectively.

### Subcellular localization

The *VvBBX44* CDS not including the termination codon was inserted into the pCAMBIA2300-eGFP vector. The resulting *VvBBX44-eGFP* and the empty vector *eGFP* control constructs were transformed into *Agrobacterium* EHA105. Tobacco leaves were used for transient transformation by infiltration with *Agrobacterium*. *Agrobacterium* containing H2B-mCherry was used to mark the nucleus. Transformed tobacco was observed using a Leica TCS SP5 confocal laser-scanning microscope 72 h after transformation.

### Luciferase reporter assay

The *VvUFGT* promoter (~2000 bp upstream of ATG), *VvMYBA1* promoter (615–5 bp upstream of ATG), and *VvBBX44* promoter (2007–1263 bp upstream of ATG) were amplified and cloned into pCAMBIA1302-LUC to generate the reporter constructs *pUFGT*-*LUC*, *pMYBA1*-*LUC*, and *pBBX44*-MRE-*LUC*, respectively. The CDSs of *VvBBX44*, *VvHY5*, and *VvMYBA1* were cloned into pSAK277 as the effector, and designated OE-*VvBBX44*, OE-*VvHY5*, and OE-*VvMYBA1*, respectively. These constructs were transformed into EHA105 *A. tumefaciens*, respectively. Then the effector and reporter pairs were co-infected into tobacco leaves by agroinfiltration, respectively. LUC was used as a reporter to detect the transcriptional activation of VvBBX44, VvHY5, and VvMYBA1. To normalize the signal between the empty vector (EV) and transient lines in the luciferase assays, we controlled the concentration of bacteria by measuring the OD_600_, marked the infected area to ensure consistent sampling location, and weighed samples of equal quality. This facilitated adjustment of the total protein content after measuring protein concentration. LUC and REN (background level) activities were detected using the Luciferase Reporter Assay kit (Promega, Madison, WI, USA). EV combined with the reporter construct was used as a negative control. At least three biological replicates were examined for each sample.

### Promoter sequence analysis

Genomic sequences extending to 2000 bp upstream of the ATG start codon in both the sense and antisense strands of *VvBBX44* and *VvMYBA1* were selected as the promoter regions. Analysis for *cis*-regulatory elements was done using the PlantCare database (http://bioinformatics.psb.ugent.be/webtools/plantcare/html/).

### Electrophoretic mobility shift assay

The cDNA sequences of *VvBBX44*, *VvHY5*, and *VvMYBA1* from ‘Jingxiu’ leaves were inserted into the pET-30a-c (+) vector (Cat. No. 69337–3) and transformed into *Escherichia coli* (DE3). His-tagged fusion proteins were purified using Ni-NTA columns from cells induced at 16°C for 16 h. The eluted protein was verified by Coomassie brilliant blue staining and western blotting. The biotin-labeled DNA probes used in the EMSA are listed in Supplementary Data Table S1. Non-labeled probes were added as competitors. The probes were heated at 95°C for 5 min and left to cool to room temperature. The assay was performed using the LightShift Chemiluminescent EMSA Kit (Cat. No. 20148, Thermo Scientific). A 20-μL reaction mixture (containing 2 nM of biotin-labeled DNA and 800 ng of purified protein) was prepared according to the kit instructions. After 30 min of incubation at 25°C, the reaction mixture was resolved by electrophoresis on a 5% polyacrylamide gel containing 3% glycerol, transferred to a positively charged nylon membrane, UV cross-linked, and evaluated for biotin signals.

### Transformation of grapevine leaves and berry

Transient transformation of grapevine leaves was performed as described [63] with some modifications. New fully spread ‘Jingxiu’ vine leaves (~20 days after emergence) were selected for vacuum infiltration. After rinsing and drying, these leaves were inserted into MS medium. The transformed grapevine leaves were cultivated in the light for 48 h after acclimation in the dark for 24 h, and then sampled.

Transformation of berries was performed as described by Xu *et al*. [63] with some modifications. *Agrobacterium* strain EHA105 carrying the Cas9 and sgRNA gene for *VvBBX44* editing were grown overnight at 28°C with shaking at 200 rpm in 1 L of Luria–Bertani liquid medium supplemented with 50 mg L^−1^ rifampin and 50 mg L^−1^ kanamycin. The bacteria were harvested by centrifugation at 3281 × g for 10 min, resuspended in induction buffer [10 mM MES pH 5.6, 10 mM MgCl_2_, and 150 μM acetosyringone] to OD_600_ = 0.6 and incubated at 28°C for 3 h. Healthy berries 14 days before veraison were detached and washed under running water. The berries were then immersed in the suspension solution, subjected to a vacuum at 0.085 MPa for 1 h, and then the vacuum was slowly released. The infected berries were rinsed in sterile water three times, allowed to dry briefly, and inserted into MS medium coated with tin foil. After 24 h in darkness, the berries were incubated in the light for 3 days. After GFP detection, individual berries were peeled and sampled; part of the samples was used for DNA extraction to confirm the presence of the mutation at the target site by PCR and sequencing, while the other part was used for qRT–PCR.

### Statistical analysis

In this study, we perform Student’s *t*-test and Duncan’s test to evaluate the significance of differences between experimental groups. The significant and extremely significant levels were defined as *P* < .05, and *P* < .01, respectively.

## Supplementary Material

Web_Material_uhad176
