# Supplementary material for: VvBBX44 and VvMYBA1 form a regulatory feedback loop to balance anthocyanin biosynthesis in grape
Source: Hortic Res. 2023 Sep 1;10(10):uhad176. doi: 10.1093/hr/uhad176 (PMC10585713; doi:10.1093/hr/uhad176)
Supplement: Web_Material_uhad176 [file web_material_uhad176.zip › Supplemental Data 1-Sequences of VvMYBA1, VaMYBA1 and VvBBX44 promoter fragments used in this study.docx]

**Supplementary Data 1. Sequences of *VvMYBA1, VaMYBA1* and *VvBBX44* promoter fragments used in this study**

>Promoter sequence fragment of *VvMYBA1* (*pMYBA1*) (615-5 bp upstream of ATG, red letters representing the T/G-box)

AGAAACAAAGTCCTCCGCGAGCCAGAGGCATATCCTAATGATTGTACCTTTCCTCACTGACAATTCCCATTAATTAGCTGCTGCCACTGCATAGCGGCTATAATATAATGGTAGAGGACCCATGGAGCTTTCCTTTTCAGTGAACATGGGTTAGTCGACAAAAGAAAATGTTCAAGTTGAAAGAGGAGCGGTGGCCCTCAAAGGTTCCCGTCACTTGGTTGCTTTTTGTCTAAGGAAACAGTGGTATCAGAATCCAAATCTTCTACGTAATGTCCCATTCATCCTACCAATGTCCATATGAATTCCTCTGGACATTAAAAACTGGTAGCACGTGGTTGTCTTCAGGATCACACCAGTTTATACATTTTGACCACAAAATAGAGATTGTTCATAAAGGATACTAGTCAGCAATTAATTCCTAAATATCTCTTATGACACACACCCTTTGTCCATGAACTCCAGCGCATTTGGAAGCCAGTAATGCACCATAAGAAACGTGTCGAATAAACCAATTAGGGGTCTGGTGTCCGAGTCATGAGATAGAACAGGTTCGAGGTTGTTATATATCAATCAATAATTAGAGAAGGAGCCGGTCTCTTGTGTTGAGTTGA

>Promoter sequence fragment of *VaMYBA1* (*pMYBA1*) (615-5 bp upstream of ATG, red letters representing the T/G-box)

AGAAACAAAGTCCTCCGCGAGCCAGAGGCATATCCTAATGATTGTACCTTTCTTCACTGACAATTCCCATTAATTAGCTGCTGCCACTGCATAGCGGCTATAATATAATGGTAGAGGACCCATGGAGCTTCCCTTTTCAGTGAACATGGGTTAGTCGACAAAAGAAAATGTTCAAGTTGAAAGAGGAGCGGTGGCCCTCAAAGGTTCCCATCACTTGGTTGCTTTTTGTC.AAGGAAACAGTGGTATCAGAATCCAAATCTTCTACGTAATG............................AATTCCTCTGGACGTTAAAAAATGGTTGCACGTGGTTGTCTTCAGGATCACACCAGTTTATACATTTGGACCACAAAATACAGATTGTTCATAAAGGATACTAGTCAGCAATTAATTCCTAAATATCTCTTATGACACACACCCTTTGTCCATGAACTCCAGCGCATTTGGAAGCCAGGTAATGCACCATAAGAAACGTGTCGAATAAACCAATTAGGGGTCTGGTGTCCGAGTCATGAGATAGAACAGGTTCGAGGTTGTTATATATCAATCAATAATTAGAGAAGGAGCCGGTCTCTTGTGTTGAGTTGA

>Promoter sequence fragment of *VvBBX44* (*pBBX44-MRE*) (2007-1263 bp upstream of ATG, red letters representing the MRE)

CCATCACTTCAAACCAAGGGAGATTCACCTATTTACTTTCCATTAATGTTCTGGTATACATCAAATCCTATGAAAACCTAAGCCATAGAGTTTCGGAGGTTGAGAAAGCCTAAGGGGATCGTGCACCACAGATTGATGAAAAACAACAAAAAGAAAAAAATATGGTAGCTGACCTTCACACCCATTTCAATCCGTGCTTATCTACCACCATCGAAAGTCTTGTTTCTTTCAAATTCTGGCCACTTGGGAAAGAAATCTAGTTATCCATGTAGTGAAGCAACTTTCTACCTGAAAATGATATGGTAGAAGAGAAAGTATATAGGATATGGACTCGTCTACTCCCAAAATTATCTAAAAGCAATCATTTTAATGAGATATTTGGGAGCAAATATGTGATGGGTACTGTAGAGCAAATCTGGGGTTACATAAAGGATTTGATATTTAGGGATAACAAACCAAAAATGGAAGGGTCCACCTGAGGAAGTTGGCAGTGGATCAAAGACAATAAGAGGCCACGTGTTGGACAACATATCTGATCTATGCGACCATAAAAGACACGTCAACCTCTGAATCCTTTCCAGGCTTTCCAGAATCCTGGCCAGCTAGCCTAAGGACTGTGAGGGCCAAGGCGCAAAAAATCGAGGAGAACAATACAAGCGCACCACTCGAGTCATCGCTCAGAGAAATATACGCGTCTCCCTTCTTTTTTTTTGGGGTAAAGTTGGTGGTTGGGCTTTGCTTCTCA
