# Supplementary figures and images for: VvBBX44 and VvMYBA1 form a regulatory feedback loop to balance anthocyanin biosynthesis in grape
Source: Hortic Res. 2023 Sep 1;10(10):uhad176. doi: 10.1093/hr/uhad176 (PMC10585713; doi:10.1093/hr/uhad176)

**A**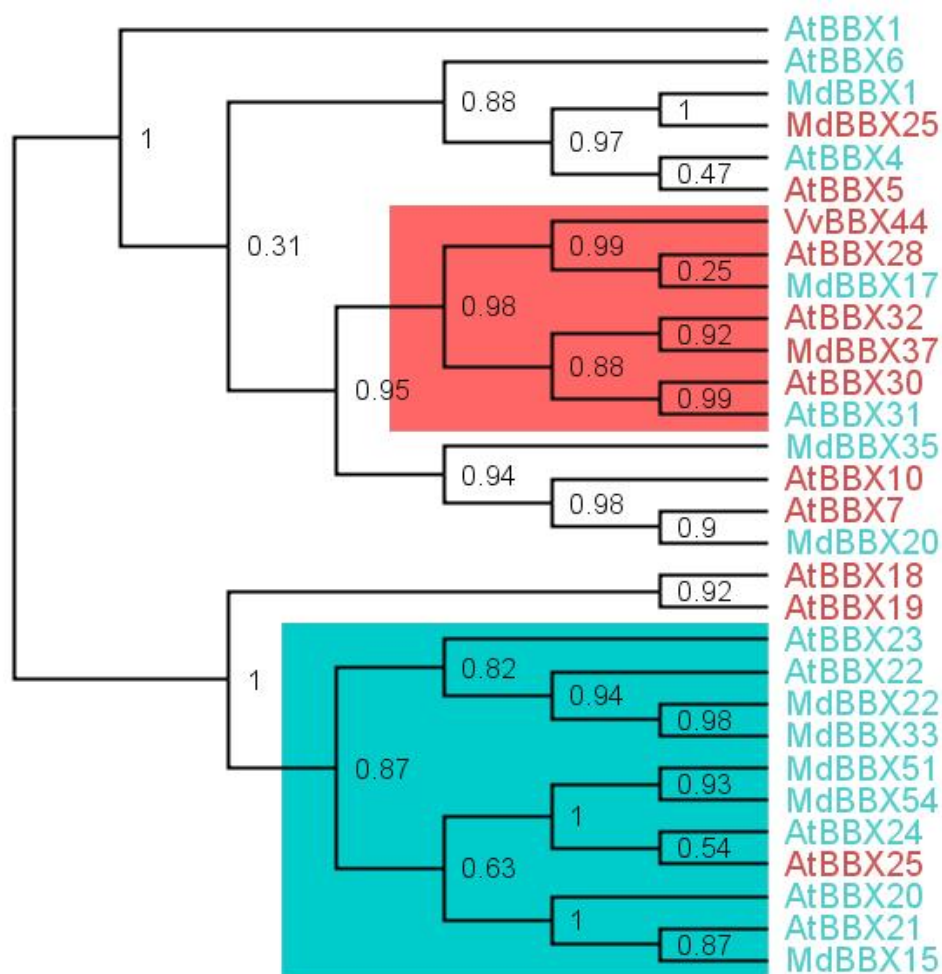**B**

Stable transgenic  
*V. amurensis* calli

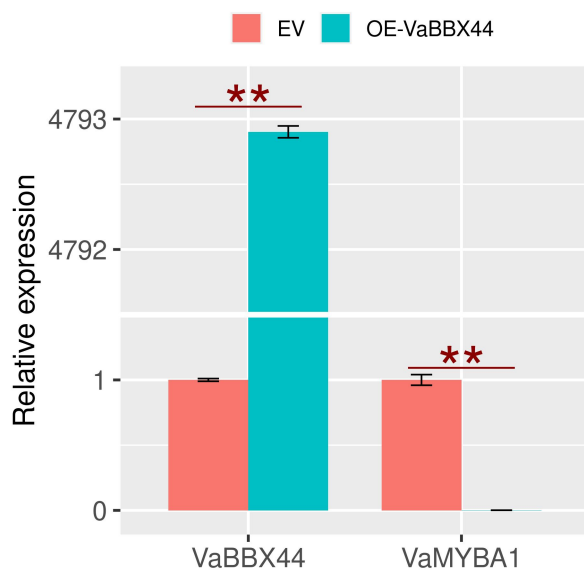**C**

Transient transgenic  
'Jingxiu' leaves

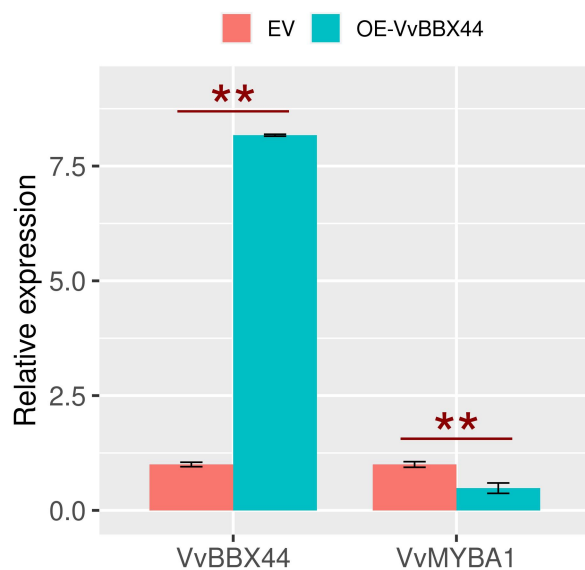

Supplement: Web_Material_uhad176 [file web_material_uhad176.zip › FigS1-2.pdf]

**A****KO-EV**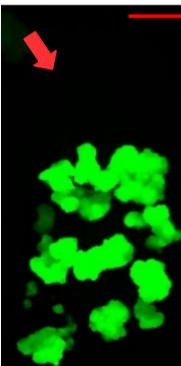**KO-VaBBX44**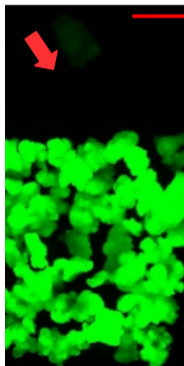**B****Dark****KO-EV**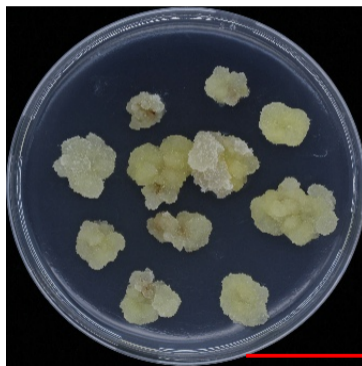**KO-VaBBX44**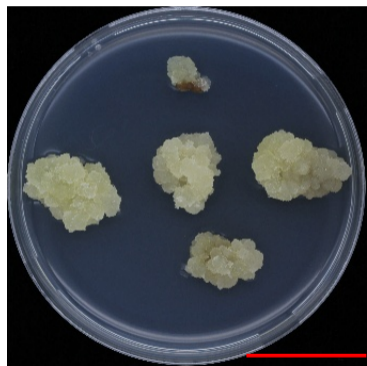

Supplement: Web_Material_uhad176 [file web_material_uhad176.zip › FigS2.pdf]

## 2012-‘Pinot noir’

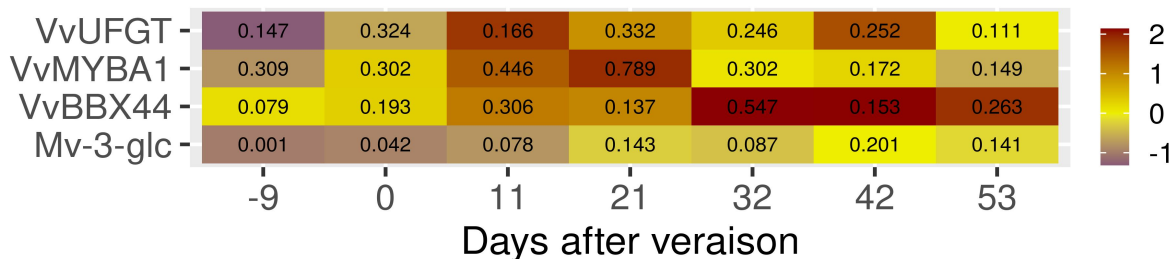

## 2013-‘Pinot noir’

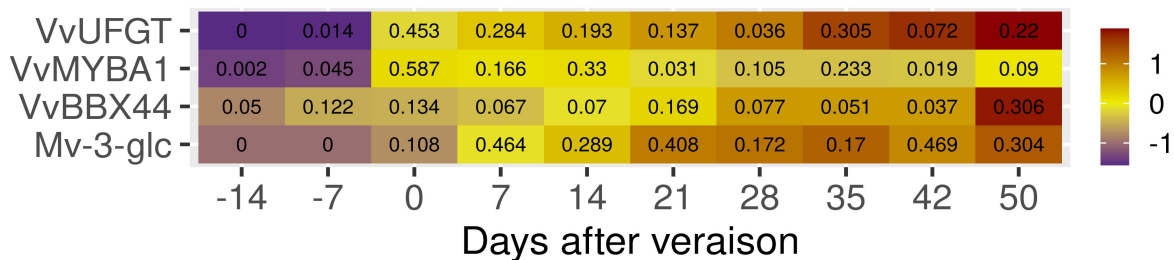

## 2014-‘Pinot noir’

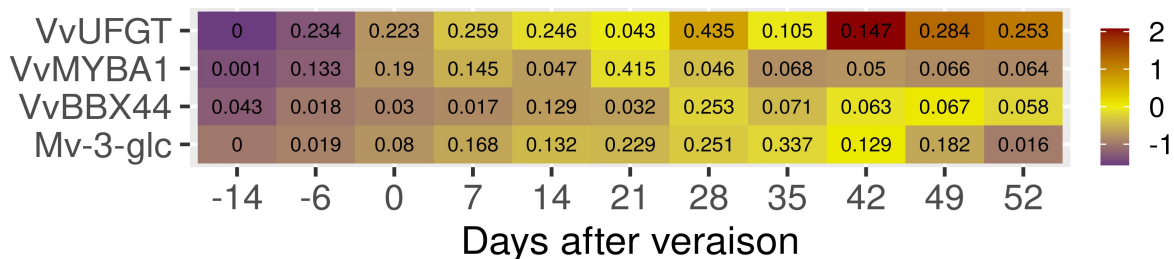

Supplement: Web_Material_uhad176 [file web_material_uhad176.zip › FigS3.pdf]

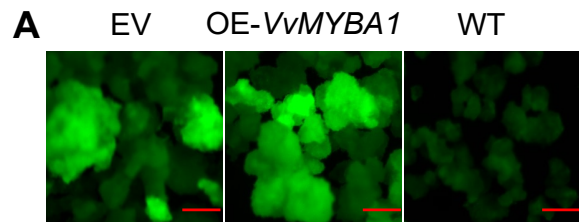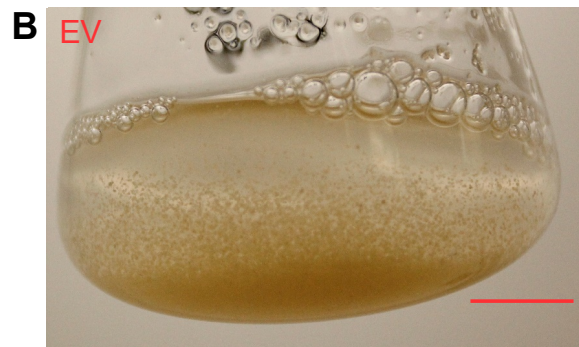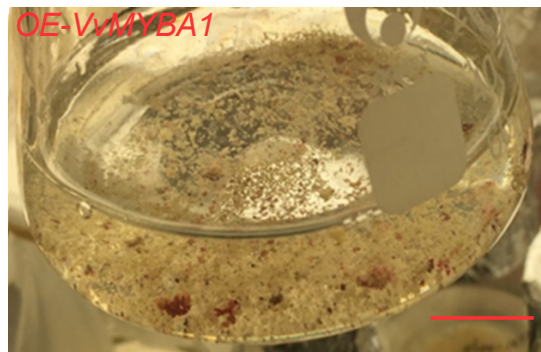

**C** Stable transgenic 41B grape calli

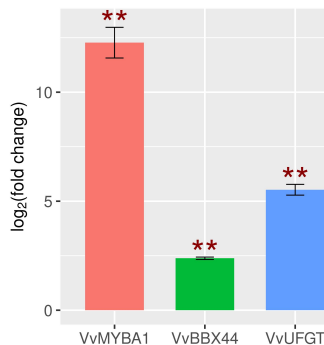

**E** Transient transgenic 'Jingxiu' berries skin

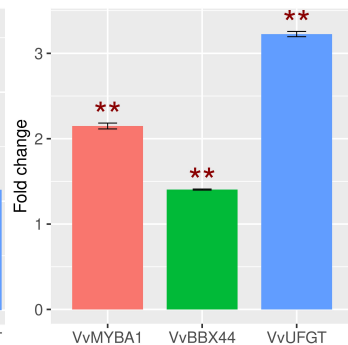

**D**

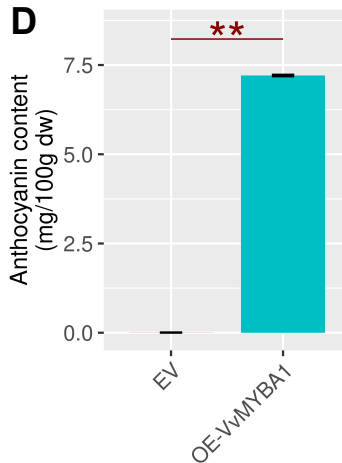

**F**

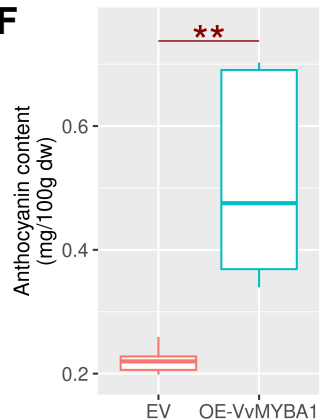

Supplement: Web_Material_uhad176 [file web_material_uhad176.zip › FigS4.pdf]

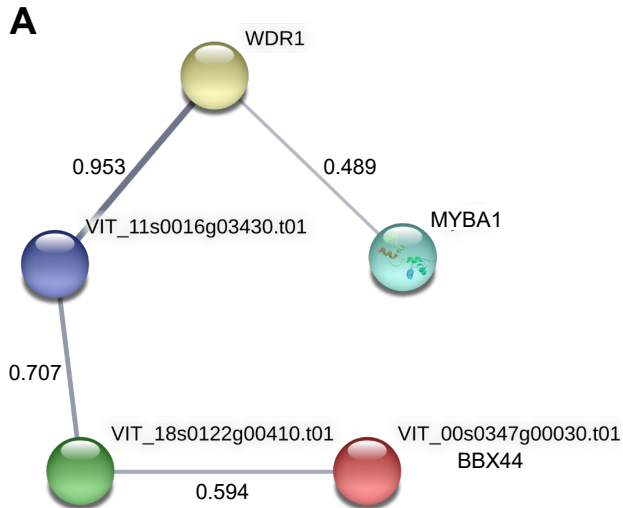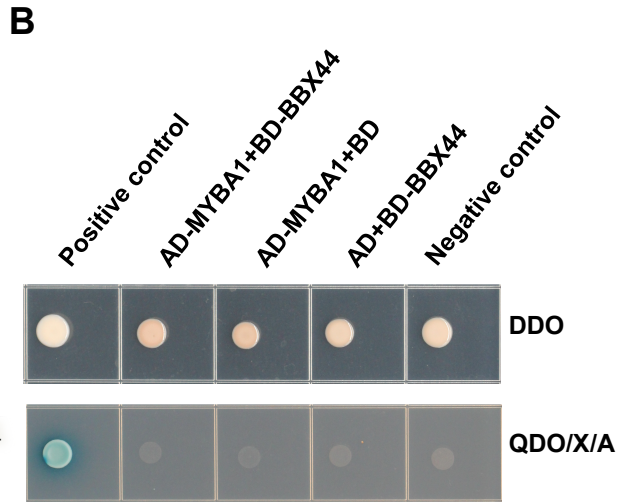

Supplement: Web_Material_uhad176 [file web_material_uhad176.zip › FigS5.pdf]

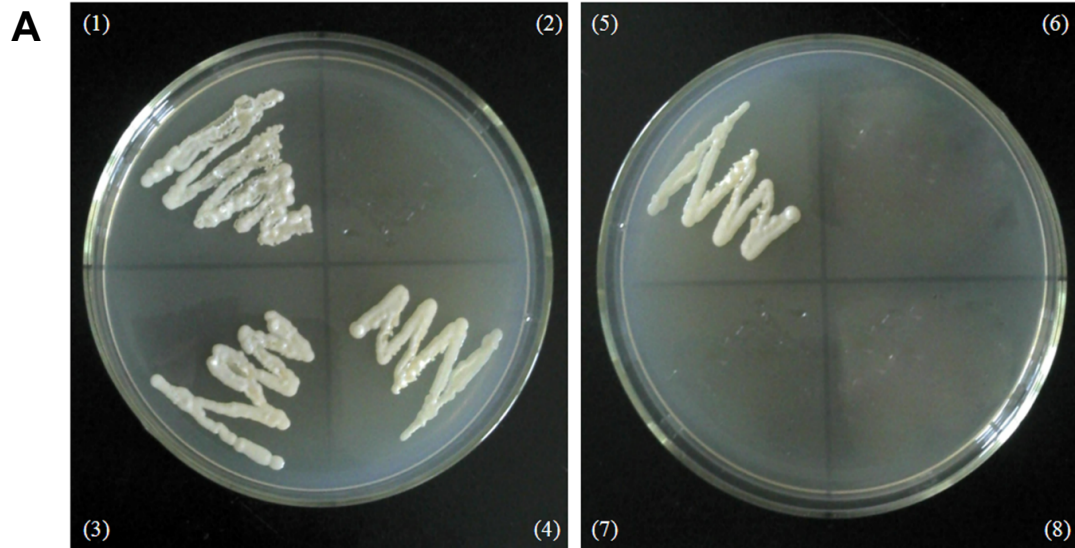

**B**

Marker  
Before purification  
After purification

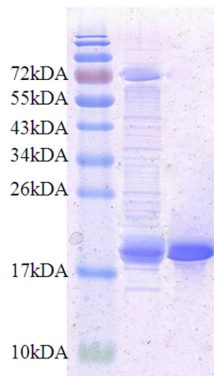

**C**

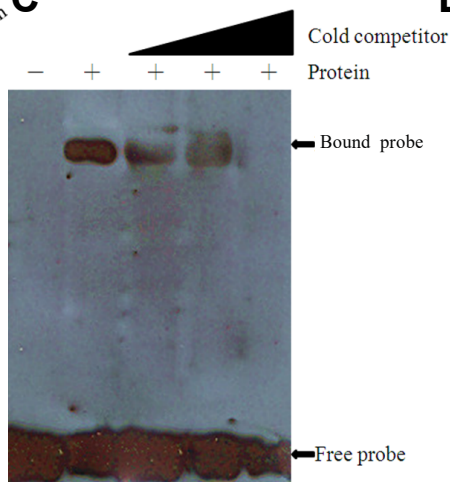

**D**

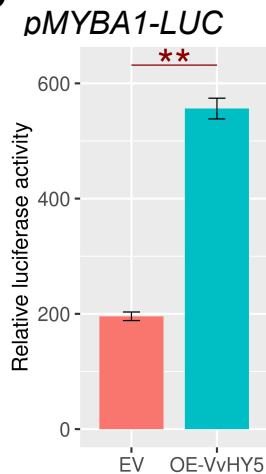

Supplement: Web_Material_uhad176 [file web_material_uhad176.zip › FigS6.pdf]
